# HIV Prevention After Discontinuing Pre-Exposure Prophylaxis: Conclusions From a Case Study

**DOI:** 10.3389/fpubh.2018.00137

**Published:** 2018-05-09

**Authors:** Kai J. Jonas, Natthakhet Yaemim

**Affiliations:** ^1^Work and Social Psychology, Maastricht University, Maastricht, Netherlands; ^2^Pulse Clinic, Bangkok, Thailand

**Keywords:** HIV, pre-exposure prophylaxis, prevention, public health, counselling

## Abstract

Pre-exposure prophylaxis (PrEP) with tenofovir disoproxil fumarate in combination with emtricitabine (FTC) is a highly effective form of HIV prevention. Endeavors of health-care providers and activists in many countries over the world are directed at making access to PrEP possible, or increasing PrEP use among men-who-have-sex-with-men (MSM). We argue while this effort is necessary, we also need to consider modes of HIV prevention after a period of PrEP use. PrEP uptake is not a one-way street, meaning that individuals may discontinue PrEP use, either voluntarily and involuntarily. Voluntary discontinued PrEP use in conjunction with decreased or no HIV risk exposure is unproblematic, but involuntary discontinuations with continuous high level of HIV risk exposure calls for tailored post-PrEP use HIV prevention. We present a case study of an MSM individual who discontinued PrEP for medical reasons (renal function) and seroconverted soon afterward, to illustrate the need for tailored HIV prevention post-PrEP. Furthermore, we provide additional contexts of PrEP discontinuation leading to populations that are in need for post-PrEP types of HIV prevention. Subsequently, we present suggestions for modes of post-PrEP HIV prevention based on knowledge–communication–choice model. Community organization and health-care providers should consider and prepare their HIV prevention consulting protocols for such types of clients and add post-PrEP HIV prevention measures to their consulting offer.

Given that pre-exposure prophylaxis (PrEP) with tenofovir disoproxil fumarate in combination with emtricitabine (FTC) is a highly effective form of HIV prevention (1–7), it is no surprise that a number of countries (e.g., USA, Belgium, France, Norway, and Thailand) have made PrEP available for HIV high risk populations, such as men-who-have-sex-with-men (MSM). The current endeavor is to increase PrEP uptake which is often still at suboptimal population levels, for example in the USA (8) or Thailand (9). In a number of other countries, medical service providers, community-based organizations (CBOs), activists, and lesbian, gay, bisexual, and transgender (LGBT) associations are lobbying governments to make PrEP available. Independent of formal availability and insurance coverage, LGBT associations and medical service providers are focusing on MSM to increase PrEP uptake, since levels are still comparatively low (8, 9).

It may seem rather unexpected at this point in time to call for a consideration of alternative modes of HIV prevention after discontinuing PrEP. Yet, we argue, it is necessary to consider this option as well, since the use of PrEP is not meant to be a lifelong form of HIV prevention, or put differently, a one-way street. Quite to the point, the World Health Organization presented PrEP use as a prevention tool to be used in “seasons of risk,” and thus conceptualized an HIV prevention without PrEP, after PrEP use (10). The notion of periods of PrEP use and non-PrEP use dovetails with event-driven PrEP, too (11). Yet this post-PrEP type of HIV prevention seems to have moved out of focus, currently, and may need to be revisited given increasing PrEP users.

Assuming that people who have discontinued PrEP will go back to condom use as their primary prevention strategy is flawed for a number of reasons. Certain PrEP users have history of condomless anal intercourse (which is a reason why they used PrEP in the first place), and thus will not go “back” to condom use, since this was not their primary HIV prevention strategy (7). Another reason is the possible decrease of condom use under PrEP, or completely cessation, which can lead to difficulties introducing condoms again. Decreased condom use under PrEP is based on norms prevalent in sexual networks, and the awareness that other STIs are treatable (12). The empirical basis supporting this is mixed. While earlier research trials did not report lower condom use (13–15), more recent studies outside of research trials report that condom use can decrease and that other STI can rise, but the picture is far from coherent (16–19). The explanation for this discrepancy may lie in different PrEP user populations.

First, we present a case of a former PrEP user who seroconverted soon after discontinuing PrEP. The case report has been approved by the Ethical Review Board of the Faculty of Psychology and Neuroscience, Maastricht University (ERCPN-180_04_06_2017). The patient reported in the case provided written informed consent (on file). Second, we will outline potential additional scenarios of discontinuation of PrEP use. Third, we are going to discuss potential avenues of post-PrEP HIV prevention.

## Case Report: HIV Infection after PrEP Use

We report a case of a 56-year-old German MSM residing in Bangkok. He moved to Thailand late in 2015, and after 2 months in Bangkok started to take PrEP. At the time of the reported period he identified as single, with an average of two partners per week, having both condom and condomless anal intercourse with his sex partners. Regular erectile dysfunction medication (Sildenafil 100 mg tablet, and jelly and prostaglandin injections) use was reported, as well as recreational drug use that included gamma-butyro-1,4-lacton (GBL/GHB), crystal methamphetamine, ecstasy regularly, and occasionally ketamine.

In January 2016, he started taking PrEP provided by Thai Red Cross, Bangkok. Follow-up renal tests were conducted at Pulse Clinic, Bangkok [31 March 2016: blood urea nitrogen BUN 16 (normal range 7–20 mg/dL), Cr 1.69 mg/dL (normal range 0.5–1.3 mg/dL), eGFR 72 (normal range for this race and age group >90); 24 May 2016: BUN 31.2, Cr 1.38 mg/dL, eGFR 84; 6 July 2016: BUN 17.6, Cr 1.33 mg/dL, eGFR 64]. On 21 November 2016, he stopped taking PrEP on medical advice as a result of the renal function results described earlier. On 23 November 2016, he was diagnosed with secondary syphilis (presented with palm and sole rash; RPR titer 1:32 and TPHA reactive, FTA-ABS IgM, IgG reactive) and was treated with benzathine penicillin 2.4 million U injection IM, once a week for 3 weeks continuously. A follow-up test conducted 19 December 2016 after completion of treatment showed an RPR titer 1:1. After syphilis treatment, a negative HIV test and renal function test (creatinine 1.26 mg/dL and eGFR 62) he never started taking PrEP again due to kidney function parameters.

On 8 January 2017, after having UAI with receptive partner who had confirmed diagnosis of syphilis proctitis, a novel episode of primary stage syphilis was detected (RPR titer 1:2 and TPHA reactive, FTA-ABS IgM reactive) and treated subsequently with benzathine penicillin 2.4 million U injection IM, single treatment, follow-up test after completion of treatment on 8 April 2017 RPR titer reactive 1:1.

On 8 April 2017, he wanted to start taking PrEP again, but renal function parameters counter-indicated PrEP use (Cr 1.53 mg/dL and eGFR of 50). While being fully aware of not being on PrEP and thus not protected against HIV, he continued to have anal sex without a condom, often engaging in “Chemsex” (recreational drug use in a sex context) and being both the receptive and insertive partner with internal ejaculation.

In May 2017, a routine HIV test (ALERE Determine HIV-1/2, DoubleCheckGold™ Ultra HIV1/2 and SD Bioline HIV1/2 3.0 showing HIV Type 1) confirmed his sero-conversion. Viral load (PLASMA HIV-1 RNA PCR ROCHE AmpliPrep/TaqMan V.2.0) was at 47,000 copies/mL and CD4 cell count at 395/mm^3^. In June 2017, antiretroviral therapy was initiated with Kivexa^®^ (abacavir/lamivudine) and Edurant^®^ (rilpivirine). As of July 2017, viral load was 231 copies/mL and CD4 count of 523 cells/mm^3^.

This case is informative in many ways. First of all, it shows an MSM who seeks to protect himself against HIV in a context of high HIV prevalence (9). His behavior prior and during using PrEP is characterized by occasional condom use. But, the case also shows that such an individual is finding it hard to downregulate condomless sex after a discontinuation of PrEP. His subsequent HIV infection speaks to this matter. Finally, awareness that he was no longer protected by PrEP was not sufficient to induce him to return to other means of HIV prevention. Before we turn to post-PrEP HIV prevention measures, we want to briefly describe other related scenarios of PrEP discontinuation.

## Other PrEP Discontinuation Scenarios

While there are a number of non-critical PrEP discontinuation scenarios, for example, a change in risk behavior (no more anal intercourse) or a stable HIV negative partner in a monogamous relationship, there are also a number of critical scenarios that can lead to continuously high levels of HIV risk exposure, but without the biomedical protection of PrEP. There is of course also a voluntary discontinuation, simply based on the decision that PrEP is not the preferred HIV prevention strategy. Yet in this case, it is safe to consider that other forms of HIV prevention are put into place, or that the exposure risk itself is discontinued. We consider such intentional discontinuation decisions non-problematic in this context, since the individual prefers other prevention strategies over PrEP. All other contexts we refer to as involuntary discontinuation due to health reasons, or lack of procurement opportunities. The scenarios listed below are clearly not applicable to all contexts (e.g., cultures, countries, health-care systems, and health insurance systems) but may apply selectively.

### Discontinuation due to Side Effects

Similar to our presented case, PrEP users may experience side effects (either on the renal function parameter level or directly experienced), which force them to discontinue PrEP.

### Discontinuation due to Lack of Funds

Individuals who had access to PrEP may lose this access due to a lack of funds. This lack of funds can be due to a lack of money to pay for PrEP (either completely, or in an insurance co-payment scheme). Generic versions of PrEP can alleviate this hurdle to a certain degree, but then generic PrEP is not always available or still too costly. For example, MSM sex workers in the low and lower-middle income countries who pay for PrEP themselves may have to refrain from getting PrEP during low income phases.

### Discontinuation due to Change of Insurance Policy

Political changes on a national level can have serious implications on insurance policies regarding the scope of coverage. The current debate on the Patient Protection and Affordable Care Act in the USA is a prime example with open outcomes. Furthermore, social mobility (upward or downward) can lead to a change of insurance programs which may lead to a loss of PrEP coverage, when moving from one insurance company to another. For example, university students can experience such contexts: While initially covered under campus insurance plans, first own health-care insurance may not cover PrEP.

### Discontinuation due to Availability or Relocation

Informal procurement of PrEP, for example, *via* buyer’s clubs, online pharmacies or PrEP tourism (picking up PrEP in a foreign country where it is available) can cease when the source dries out (e.g., due to lack of travel opportunities).

Individuals using PrEP that move out of a country where PrEP is available formally may find themselves in a context where they cannot rely on their PrEP HIV prevention strategy, simply due to unavailability. Especially if PrEP access (often determined by insurance coverage) in their country of departure ceased, these individuals may find themselves in a vacuum of PrEP access (e.g., professionals moving out of the USA to a European country where PrEP is not available).

## HIV Prevention after PrEP

When an MSM discontinues PrEP, and the HIV risk exposure does not decrease at the same time, alternative HIV prevention is needed. If condom use has decreased or fully ceased while using PrEP (due to newly formed habits), it is not coming back instantly after a discontinuation of PrEP. Second, not all PrEP users were using condoms before, thus going back to condom use is not a viable strategy at all. MSM in a post-PrEP phase need to be provided with a combination of alternative HIV prevention strategies. Those strategies may differ in HIV protection levels, but individuals need to be encouraged to combine strategies that work best for their needs, their selection of sex partners and HIV risk exposure. This suggested knowledge–communication–choice approach (KCC) goes beyond mere informed choice:

- *Knowledge* about all alternative HIV prevention possibilities
- *Communication* skills to address HIV status and prevention options with sex partner(s)
- *Choice* behavior, i.e., the ability to make and maintain informed choices regarding risk practices and sex partners

Men-who-have-sex-with-men using PrEP and/or condoms as their HIV prevention strategies can base their strategy mainly on the last element of the KCC approach, namely, choice behavior. Individuals who discontinued PrEP now lack a choice option and therefore need to combine all three elements to achieve optimal protection. They need to be educated about their remaining options, empowered to talk openly about it and to make sex partner(s) or behavior choices that fit their HIV prevention strategy.

In particular, we suggest a combination of the following considerations:

- PrEP use of sex partner—knowledge about such biomedical prevention behavior in third parties, open communication about it and partner choice. While this third party-driven strategy not providing optimal levels of protection, it still offers some levels of vicarious protection, given adequate PrEP adherence/HIV negative status of the PrEP-using sex partner(s)
- HIV status of sex partner(s) (sero-sorting)—knowledge of the HIV status of sex partner(s) and communication about their own discontinuation of antiretroviral-based HIV prevention
- Treatment as prevention—as knowledge about the effectiveness of this strategy and limiting condomless sex to HIV-positive partners with undetectable viral load
- Condom use for anal intercourse—choice of prevention strategy
- HIV status—knowledge about regular HIV testing and actual status
- High risk practices—knowledge about the HIV risk associated with different practices [e.g., lack of control during Chemsex (20)] and knowledge about HIV risk-reduction strategies (e.g., avoidance of internal ejaculation in the context of being the receptive partner in condomless anal intercourse, or rejection of condomless anal intercourse in specific contexts such as sex work or transactional sex)
- High risk STI-HIV contexts—knowledge that bacterial STIs can increase HIV infection risks

Clearly, not all strategies apply to all clients who discontinued PrEP. Medical service providers and HIV/STI counselors should adopt a pragmatic approach to determine and discuss the optimal combination of prevention measures for clients who discontinued PrEP and revisit such strategies regularly. CBOs, LGBT, and social service providers need to support people who discontinued PrEP to create a social norm in which their tailored HIV prevention strategy is getting recognized and accepted. In a nutshell, people who discontinued PrEP need special counseling and empowerment to navigate safely in their PrEP-less world.

The introduction of PrEP has led to a focus on PrEP uptake. What has been missed under this perspective is an HIV prevention strategy for MSM who discontinue PrEP. To assume that they will “go back” to condom use is often unrealistic. Instead, a combination of HIV prevention strategies needs to be discussed to find the set that serves their needs best. Post-PrEP HIV prevention is a phenomenon that can affect MSM of all cultural backgrounds, high and low SES status, and ages. Our suggested KCC approach is a selection of knowledge- and behavior-based factors that can decrease HIV infection risks. The considerations differ in effectiveness, but even vicarious PrEP use can, in the absence of other forms of HIV prevention, be a relevant contribution to HIV prevention. Essential is the awareness of individuals who discontinued PrEP that they are much more vulnerable than before. Otherwise, we fear, the risk of post-PrEP HIV infections will increase, similarly to the case we presented.

## Ethics Statement

This study was carried out in accordance with the recommendations of “Code of Ethics for Research in the Social Sciences involving Human Subjects and other national, European and international codes and guidelines for scientific research involving human subjects, Ethical Review Committee of the Faculty of Psychology and Neuroscience of Maastricht University, The Netherlands” with written informed consent from all subjects. All subjects gave written informed consent in accordance with the Declaration of Helsinki. The protocol was approved by the “Ethical Review Committee of the Faculty of Psychology and Neuroscience of Maastricht University, The Netherlands,” code ERCPN-180_04_06_2017.

## Author Contributions

KJ and NY wrote the manuscript and analyzed and interpreted the case report. NY collected the case report data.

## Conflict of Interest Statement

The authors declare that the research was conducted in the absence of any commercial or financial relationships that could be construed as a potential conflict of interest.
